# *De novo* whole-genome assembly of the *Wolbachia* sp. endosymbiont from *Anastrepha fraterculus* using long- and short-read metagenomic data

**DOI:** 10.1128/mra.00425-26

**Published:** 2026-04-23

**Authors:** Claudia A. Conte, Maximo Rivarola, Sergio Gonzalez, Fabian H. Milla, Celeste Soria, María C. Giardini, Diego F. Segura, Alfred M. Handler, Kostas Bourtzis, Jiannis Ragoussis, Silvia B. Lanzavecchia

**Affiliations:** 1 Laboratorio de Genética y Genómica de Insectos, Instituto de Genética, Instituto Nacional de Tecnología Agropecuaria 42651, Hurlingham, Buenos Aires, Argentina; 2 Apolo Biotech-CONICET, Santa Fe, Argentina; 3 Instituto de Agrobiotecnología y Biología Molecular-CONICET, Instituto Nacional de Tecnología Agropecuaria 42651, Buenos Aires, Argentina; 4 Instituto de Genética, GV-IABIMO, INTA-CONICET, Hurlingham, Argentina; 5 U.S. Department of Agriculture, Agricultural Research Service, Center for Medical, Agricultural, and Veterinary Entomology 57779, Gainesville, Florida, USA; 6 Department of Nuclear Sciences and Applications, Insect Pest Control Section, Joint FAO/IAEA Centre of Nuclear Techniques in Food and Agriculture, International Atomic Energy Agency 17111, Vienna, Austria; 7 McGill Genome Centre, McGill University 5620, Montreal, Canada; Indiana University Bloomington, Bloomington, Indiana, USA

**Keywords:** *Wolbachia*, whole-genome assembly, fruit fly

## Abstract

A whole-genome assembly and annotation of *Wolbachia* sp. infecting *Anastrepha fraterculus* sp. 1 were generated by a metagenomic analysis of sequencing reads from a host genome project. This study contributes to the characterization of this endosymbiotic bacterium and provides valuable insights for research on host-symbiont interactions and pest management strategies.

## ANNOUNCEMENT

Microorganisms, including archaea, bacteria, viruses, and fungi, play crucial roles in insect digestion, nutrition, development, and immunity (1). Particularly, studying symbiotic bacteria in insect pests opens new research avenues for developing innovative and sustainable control strategies (2). The South American fruit fly, *Anastrepha fraterculus* Wiedemann 1830 (Diptera: Tephritidae), is one of the most destructive insect pests in South and Central American countries causing significant economic losses (3). We present a *de novo* genome assembly and annotation of *Wolbachia* sp. infecting *A. fraterculus* Brazilian 1 morphotype from Argentina based on metagenomic analyses combining Oxford Nanopore Technologies (ONT) and Illumina-MGI reads from the host genome project (4).

Genomic DNA was obtained from a newly emerged female of the *A. fraterculus* line infected with the *w*AfraCast2_A *Wolbachia* strain (5), as previously described (4). Size selection was performed using the SRE-XS Kit (Pacific Biosciences, CA). Libraries were prepared with the SQK-LSK110 Kit (ONT, UK), sequenced on a PromethION R9 flow cell for 72 h, and base-called with Guppy-v6.4.6 (ONT, UK) in high-accuracy mode. Illumina-compatible libraries were prepared by Covaris shearing and NxSeq AmpFREE Low-DNA-Ligation Kit (BioSearch Technologies, CA, USA) with IDT xGen-Dual-Index-Adapters, converted to MGI format, and sequenced on a DNBSEQ-G400 (MGI Tech, USA) with 150-bp paired-end reads using cPAS chemistry. All sequencing services were provided by the McGill Center (Montreal, Canada). Quality control of ONT reads was conducted using Nanoplot-v1.44.0 (6), Porechop-v0.2.4 (7) to trim adapters, and Filtlong-v0.2.1 (8) to filter long reads. A *de novo* metagenome assembly was achieved with Flye-v2.9.5-b1801 (9) using the --meta option. Kraken2 with the StandardPF database (10) was used for contig taxonomic classification. The contig assigned to the *Wolbachia* taxon was extracted using Seqtk-v1.5 (11), and terminal overlap was verified by self-alignment with Minimap2-v2.3 (12). Illumina-MGI short reads were used for polishing using Pilon-v1.24 (13). Genome completeness and integrity were evaluated with Benchmarking Universal Single-Copy Orthologs (BUSCO)-v5.8.2 (14) and the *Rickettsia* gene data set. Pairwise average nucleotide identity (ANI) was calculated using FastANI-v1.34 (15) against the *Wolbachia* endosymbiont of *Drosophila melanogaster w*Mel (NZ_CP046925.1). Structural annotation was performed with RASTtk-v1.073 (16). The *dnaA* gene position was determined from the annotation, and the sequence was rotated using SeqKit-v2.10.0 (17). PHASTER (18) was used to annotate prophage sequences within the *Wolbachia* genome.

**Fig 1:**
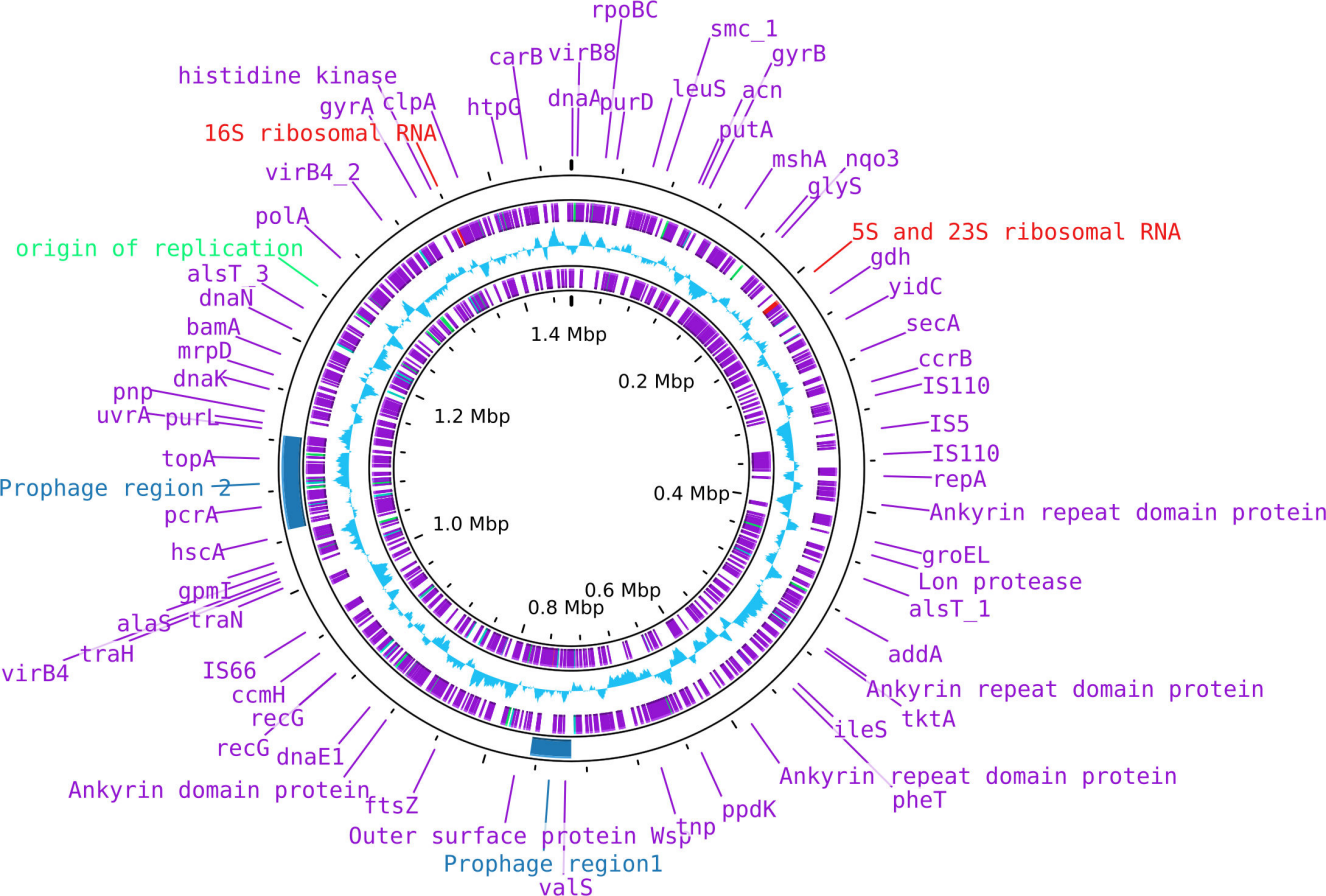
Representation of the genome of the *Wolbachia* strain *w*AfraCast2_A infecting *Anastrepha fraterculus* sp. 1. From outer to inner rings: complete prophage regions, coding sequences, GC content, and reverse-strand coding sequences. The origin of replication is indicated in green, and rRNA transcription units in red.

A total of 247,588 high-quality long ONT reads were assigned to microorganisms (Table 1), and 1,363 contigs were assembled. A circular contig of 1,463,312 nucleotides was taxonomically assigned to *Wolbachia* sp. with 869× coverage. After polishing using 965,855 Illumina-MGI reads, the assembly yielded a 1,463,854 bp genome of 35.07% guanine-cytosine (GC) content and 99.1% ANI to the *wMel* reference genome. BUSCO analysis indicated a high-quality assembly with 99.5% completeness and 0.3% duplicated sequences. The annotated genome includes 1,549 predicted protein-coding sequences, 34 tRNAs, and a complete set of rRNA genes in two unlinked transcription units (16S and 23S-5S). Two complete prophage regions and 119 open reading frames (ORFs) related to insertion sequences (ISs) from IS5 and IS4 families were identified (Fig. 1). The *Wolbachia* endosymbiont of *A. fraterculus* genome provides a valuable resource for studying host-symbiont interactions enhancing pest management strategies.

**TABLE 1:** Taxonomic identification of microorganisms in female *A. fraterculus* sp.1 ONT long reads (total raw reads = 15,810,325; high-quality reads = 15,810,276)

Microorganism group	Taxonomic classification	# of reads
Virus	*Herpesvirales*	19
	*Poxviridae*	13
	*Baculoviridae*	10
	*Caudovirales*	4
	dsDNA viruses, no RNA stage	3
	Total virus	49
Fungi	*Fusarium*	4,948
	*Yarrowia*	928
	*Candida*	374
	*Kluyveromyces*	169
	*Naumovozyma*	137
	*Tetrapisispora*	135
	*Botrytis*	108
	Total fungi	6,799
Bacteria	*Wolbachia*	239,236
	*Flammeovirga*	774
	*Erwinia*	280
	*Streptomyces*	126
	*Segatella*	84
	*Staphylococcus*	51
	*Vibrio*	48
	*Sphingobacterium*	44
	*Rickettsia*	38
	*Acinetobacter*	34
	*Brevibacterium*	25
	Total bacteria	240,740
Total		247,588
Contaminants	*Homo sapiens*	82,520

## Data Availability

The raw sequencing reads have been deposited in the Sequence Read Archive (SRA) under accession numbers SRR30230720–SRR30230729 for ONT data and SRR30233774 for Illumina-MGI reads. The genome assembly of the Wolbachia endosymbiont of A. fraterculus has been deposited in GenBank accession number CM143105.1. The Whole Genome Shotgun project has been deposited in accession number JBSXML000000000 and JBSXML010000001.
